# Contractions of the Fingers and Hammer-Toe

**Published:** 1893-03

**Authors:** 


*Contractions of the Fingers and Hammer-Toe.* By WILLIAM ADAMS, F.R.C.S. Eng. Second edition. Pp. xx., 154. London: J. & A. CHURCHILL. 1892.

The author's views on the pathology and treatment of Dupuytren's contraction of the fingers are now well known, in consequence of various communications which he has made to the profession at different times, and they may be said to hold the field. The present work brings together the essays on this subject, and likewise some practical observations on the treatment of hammer-toe and of depressed cicatrices. The whole forms a useful guide to the surgeon who has to deal with these troublesome affections, and may be consulted with much advantage.

